# Lung cancer cell derived sEVs enhance the metastasis of non-small cell lung cancer via SNHG12/miR-326/SLC7A11 axis

**DOI:** 10.1080/15384047.2025.2510041

**Published:** 2025-05-26

**Authors:** Yiqian Liu, Ling Zhang, Jian Wang, Jiali Xu, Jing Xu, Mengyan Xie, Rong Wang

**Affiliations:** a Department of Oncology, The First Affiliated Hospital of Nanjing Medical University, Nanjing, China; b Department of Oncology, Jintan Hospital Affiliated to Jiangsu University, Changzhou, China; c Department of Oncology, Wuxi Second Geriatric Hospital, Wuxi, Jiangsu, China

**Keywords:** Non-small cell lung cancer, sEVs, SNHG12, metastasis, ferroptosis

## Abstract

Abnormally expressed long non-coding (lnc)RNAs are closely associated with the pathogenesis of non-small cell lung cancer (NSCLC); thus, the present study aimed to investigate the potential role of SNHG12 in NSCLC. Transmission electron microscopy and nanoparticle tracking analysis were conducted to verify NSCLC cell-derived small extracellular vesicles (sEVs). MicroRNA (miRNA/miR) and mRNA expression levels were determined using reverse transcription-quantitative PCR, while protein expression levels were determined using western blot analysis and immunofluorescence. In addition, potential binding sites between miR-326 and SNHG12/SLC7A11 were verified using a dual-luciferase reporter assay. Cell behavior was detected using flow cytometry, colony formation, wound healing and Transwell assays, and xenograft experiments were conducted to confirm the roles of SNHG12 in NSCLC. H&E staining was used for histological analysis, and each experiment was repeated three times. Results of the present study demonstrated that NSCLC-derived SNHG12 promoted type-2 tumor-associated macrophage (TAM2) polarization. However, the decrease of SNHG12 expression in EVs reduced TAM2 polarization, weakened NSCLC cell proliferation, migration and invasion, and promoted tumor cell ferroptosis. Moreover, results of the present study revealed that SNHG12 knockdown markedly suppressed tumor growth and the metastasis of NSCLC. In addition, SNHG12 upregulated SLC7A11 expression via binding to miR-326. Overexpressed SLC7A11 promoted tumor aggressiveness and suppressed the ferroptosis of NSCLC cells. Collectively, results of the present study revealed that SNHG12 suppressed ferroptosis and promoted the metastasis of NSCLC, further demonstrating that high SNHG12 expression levels may be indicative of poor clinical outcomes for patients with NSCLC. Thus, the present study highlighted that the SNHG12/miR-326/SLC7A11 axis may exhibit potential as a novel target for the treatment of NSCLC.

## Introduction

Non-small cell lung cancer (NSCLC) is the major subtype of lung cancer, characterized by high morbidity and mortality rates.^1^ Although research has focused on the development of novel treatment options, the long-term survival rate of patients remains at < 15%.^2^ Notably, this may be due to a lack of early diagnosis and distant metastasis.^3,4^ Thus, further investigations are required to identify sensitive biomarkers and determine the molecular mechanisms underlying the metastasis of NSCLC.

Long non-coding (lnc)RNAs are endogenous RNAs^5^ that play a key role in post-transcription. Moreover, lncRNAs participate in regulating cellular functions via the formation of complex regulatory networks in cells.^6^ Previous studies have demonstrated the ceRNA functions of lncRNAs. For example, lncRNA PRNCR1 enhanced the proliferation of breast cancer cells and suppressed apoptosis.^7^ In addition, lncRNA MALAT1 inhibited cisplatin sensitivity of NSCLC through regulating the microRNA/miRNA (miR)-197-3p/Catenin axis.^8^ Notably, abnormally expressed lncRNAs are closely associated with the pathogenesis of cancers, including NSCLC. LINC01123, a c-Myc-activated lncRNA, is associated with poor overall survival rates of patients with NSCLC, promoting tumor growth via sponging of miR-199a-5p.^9^ SNHG12 is a newly discovered LncRNA in recent years, which plays a certain role in the proliferation and invasion of various malignant tumors.^10,11^ There are a few reports that SNHG12 is a biomarker related to metastasis and prognosis of lung adenocarcinoma,^12^ which can mediate the proliferation and invasion of non-small cell lung cancer,^13^ regulate tumor metastasis and epithelial-mesenchymal transition,^14^ and participate in the molecular mechanism of immune escape of non-small cell lung cancer.^15^ Notably, SNHG12 is frequently upregulated in cancers, however, the specific molecular mechanism remains to be fully elucidated.

SLC7A11 is the key regulator of cystine/glutamate antiporter activity in system xc−,^16^ promoting the uptake of extracellular cystine and the synthesis of glutathione (GSH). Subsequently, GSH is used by GPX4 to reduce lipid hydroperoxides and reactive oxygen species, which suppresses lipid peroxidation and cell ferroptosis. SLC7A11 acts as an oncogene and therapeutic target in various cancers.^17,18^ Results of a previous study demonstrated that Erastin- or imidazole ketone erastin-mediated SLC7A11 deficiency induced cell ferroptosis. In NSCLC, high levels of SLC7A11 are associated with advanced stage of disease and poor 5-year survival rates of patients.^19^ Moreover, SLC7A11-mediated metabolic reprogramming promoted the malignant behavior of NSCLC cells.^20^ Results of a further previous study revealed that inhibition of SLC7A11 reduced the chemoresistance of NSCLC to cisplatin;^21^ however, the specific regulatory mechanism underlying SLC7A11 in NSCLC remains to be fully elucidated.

The present study aimed to determine the potential role of SNHG12 in NSCLC. As high SNHG12 expression levels are associated with poor clinical outcomes in patients with NSCLC; we hypothesized that SNHG12 may function as an oncogene in NSCLC. Thus, cellular functions were determined via flow cytometry, colony formation, wound healing and Transwell assays. In addition, a dual-luciferase assay was carried out to confirm the potential binding sites predicted by StarBase. Collectively, results of the present study revealed that the NRF2/SNHG12/miR-326/SLC7A11 axis may act as a novel therapeutic target in patients with NSCLC.

## Materials and methods

### Specimens

Tumor tissues and adjacent healthy tissues (≥5 cm distance from tumor tissues) were collected from 42 patients with NSCLC, who were diagnosed at The First Affiliated Hospital of Nanjing Medical University from June 1, 2020 to May 31, 2021. Following surgery, tissues samples were immediately stored at −80℃ in liquid nitrogen. Histological analysis was performed using H&E staining. The present study was approved by the Ethical Committee of The First Affiliated Hospital of Nanjing Medical University, and all patients provided informed consent.

### Cell culture and transfection

NSCLC cell lines; namely, A549 and H1299, healthy human lung epithelial cell line; namely, HBE, and THP1 cells were obtained from The American Type Culture Collection. Cells were cultured in DMEM containing 10% FBS. When 80% confluence was reached, cells were used in subsequent experiments.

Short-hairpin (sh)RNA SNHG12, sh negative control (shNC), SLC7A11 overexpression plasmids and the corresponding empty vector [small-interfering (si)RNA-NC] were purchased from GenePharm, Inc. Cells were seeded at a density of 2 × 10^4^ cells/well in a 96-well plate. Following seeding for 24 h, cells were transfected using Lipofectamine® 2000. Complexes were prepared at a ratio of 2 μl Lipofectamine® 2000 per 1 μg mRNA. mRNA was diluted at a ratio of 1:20 in H_2_O, and Lipofectamine® 2000 was diluted at a ratio of 1:10 in serum-free MEM. mRNA was added to the Lipofectamine® 2000 solution and incubated for 20 min at room temperature. The final concentration of the mRNA/Lipofectamine® 2000 solution was 25 ng/μl, and a serial dilution of 1:2 was created. In total, 10 μl of the complex solution was added to the cells and incubated for 16 h at 37℃ in 5% CO_2_. Following transfection, cells were incubated for 48 h.

In addition, macrophages were co-cultured with NSCLC cells to simulate sEV-mediated intercellular communication. Macrophages and NSCLC cells were co-cultured in CO_2_-independent medium supplemented with 0.5 mm L-glutamine and 2.5 g/l of D-glucose for 24 h, prior to incubation with or without 50 μM etoposide for 16 h. NSCLC cells were transfected with Cy3-labeled RNA.

### sEVs extraction and identification

When ≥80% confluence was reached, A549 and H1299 cells were collected and cultured in DMEM without FBS for 48 h. Subsequently, cell supernatants were collected and filtered using a PDVF membrane, and ultra-centrifugated at 120,000 × g. EsEVs were identified using transmission electron microscopy (TEM) and analyzed using nanoparticle tracking analysis (NTA) software (version, 2.3). Plasma sEVs RNA was collected using the SeraMir™ RNA Amplification kit (SBI) and calculated using reverse transcription-quantitative (RT-q)PCR.

### Macrophage induction from monocytes

THP1 cells were cultured in a 24-well plate with DMEM containing 10% FBS and 100 ng/ml PMA. Following culturing for 5 days, non-adherent cells were removed. Subsequently, macrophages were incubated in fresh medium or NSCLC-derived sEVs for 24 h.

### Malonaldehyde (MDA) and glutathione (GSH) determination

To determine the potential effects of SNHG12 on the ferroptosis of NSCLC cells, MDA and GSH release were investigated using the corresponding commercial kits (cat. nos. S0131 and S0053; Beyotime Institute of Biotechnology), according to the manufacturer’s protocols.

### Western blot analysis

Following transfection, cells were harvested and lysed, and total protein was extracted. Total protein was quantified using a BCA kit (Beyotime Institute of Biotechnology), and 30 µl protein/lane was separated via SDS-PAGE on a 12% gel. Separated proteins were transferred onto a PVDF membrane and blocked using 5% skimmed milk. Membranes were incubated with primary antibodies against CD9 (1:1,000; cat. no. ab236630; Abcam), TSG101 (1:1,000; cat. no. ab125011; Abcam), GPX4 (1:1,000; cat. no. ab125066; Abcam) and GAPDH (1:3,000; cat. no. ab9485; Abcam). Following primary incubation, membranes were incubated with secondary antibodies (1:5,000; Beijing Zhongshan Jinqiao Biotechnology Co., Ltd.). Protein bands were visualized using an ECL system (Thermo Fisher Scientific, Inc.) and analyzed using ImageJ (National Institutes of Health).

*RT-qPCR*. RNA was extracted from cells, and total RNA was reverse transcribed into cDNA using the QuantiTect Rev Transcription kit (Qiagen GmbH). qPCR was conducted using the QuantiNova SYBR Green PCR kit (Qiagen GmbH). The following primer pairs were used for qPCR: SNHG12 forward, 5’-GTGATACTGAGGAGGTGAG-3’ and reverse, 5’-CCTTCTGCTTCCCATAGAG-3’; miR-326 forward, 5’-GCCGAGCCTCTGGGCCCTTC-3’ and reverse, 5’-CAGTGCAGGGTCCGAGGTAT-3’; SLC7A11 forward, 5’-GGTGGTGTGTTTGCTGTC-3’ and reverse, 5’- GCTGGTAGAGGAGTGTGC-3’; AREG forward, 5’-GCTGAGGACAATGCAGGGTA-3’ and reverse, 5’-GTGACAACTGGGCATCTGGA −3’; IL-10 forward, 5’- GACTTTAAGGGTTACCTGGGTTG-3’ and reverse, 5’- TCACATGCGCCTTGATGTCTG-3’; CD206, forward, 5’- GTCTGAGTGTACGCAGTGGTTGG-3’ and reverse, 5’- TCTGATGATGGACTTCCTGGTAGCC-3’; FIZZ1 forward, 5’- AGGTCAAGGAACTTCTTGCCAATCC-3’, and reverse, 5’- AAGCACACCCAGTAGCAGTCATCCC-3’; and β-actin, forward, 5’-CCTGGCACCCAGCACAAT-3’ and reverse, 5’-GGGCCGGACTCGTCATAC-3’. The following thermocycling conditions were used for qPCR: A total of 40 cycles at 95°C for 22 sec, 56°C for 22 sec and 73°C for 22 sec. mRNA levels were quantified using the 2^−ΔΔCq^ method and normalized to the GAPDH gene.

### Dual-luciferase reporter assay

miR-326 binding sites on SNHG12/SLC7A11 were predicted using StarBase (version, 3.0; https://starbase.sysu.edu.cn/index.php). Wild-type (WT) or mutant (MUT) SNHG12/SLC7A11 fragments containing the binding sites were cloned into the psiCHECK2 luciferase reporter vector, and these were designed and synthesized by Guangzhou RiboBio Co., Ltd. Subsequently, cells were transfected with WT or MUT SNHG12/SLC7A11, and miR-326 mimics or miR-326 NC mimics. Following incubation for 48 h, luciferase activity was determined using a commercial kit (Promega Corporation) and calculated using RT-qPCR.

### Immunofluorescence

Cells were fixed with 4% paraformaldehyde and permeabilized with 0.2% Triton X-100. Subsequently, all cells were incubated with 5% bovine serum albumin (BSA), and primary antibodies against CD68 (1:1,000; cat. no. ab283654; Abcam) and CD206 (1:1,000; cat. no. ab64693; Abcam). Following primary incubation, cells were incubated with the secondary antibody (1:5000; Zhongshan Jinqiao Biotechnology Co. Ltd.) and counterstained using DAPI. Cells were visualized using an immunofluorescence microscope (Zeiss GmbH).

### Wound healing assay

Cells were seeded in a 24-well plate, and a scratch was created using a 100-μl pipette tip. Following 24-h incubation, cells were visualized using a microscope. Migrated cells were analyzed using ImageJ (National Institutes of Health) and values were normalized to the control cells.

### Transwell assay

Following transfection, cells were collected and resuspended. A total of 1 × 10^6^ of cells (100 µl) were seeded in the upper chamber of a 24-well plate that had been pre-coated with or without Matrigel (BD Biosciences). Subsequently, cells were incubated with culture medium containing 12% FBS for 24 h, and cells in the upper chamber were removed. Cells in the lower chamber were fixed with 4% paraformaldehyde and stained with 0.1% crystal violet. Cell migration or invasion were visualized using a microscope (Nikon Corporation).

### Colony formation assay

Following transfection, 1 × 10^6^ cells were plated in a 24-well plate. Following culturing for two weeks, cells were fixed and stained with 0.1% crystal violet, and colonies were visualized using a microscope.

### Flow cytometry

Cells were digested, lysed and filtered, and incubated with mouse Fc receptor blocker. Subsequently, cells were incubated with the anti-CD206 primary antibody (eBioscience; Thermo Fisher Scientific, Inc.) in the dark. Following primary incubation, cells were stained with intracellular antibodies, and analyzed using flow cytometry (BD Biosciences). Notably, tumors analyzed in the present study were derived from transplanted primary cells.

### PI staining

Following transfection, cells were collected and centrifuged, and stained using PI reagent. PI-positive cells were visualized using a fluorescence microscope, and the number of PI-positive cells was normalized to the number of nuclei previously determined using DAPI staining.

### TUNEL assay

Following transfection, cells were collected and fixed using 4% paraformaldehyde. Cells were permeabilized and cultured with fluorescein-labeled dUTP solution. Subsequently, cell nuclei were counterstained with 2 µg/ml DAPI, and TUNEL-positive cells were visualized using a fluorescence microscope.

### Xenograft assay

A total of 24 BALB/c nude mice (male; age, 6–8 weeks; weight, 18–22 g) were purchased from the Experimental Animal Center of Nanjing Medical University. Mice were housed under standard laboratory conditions (22 ± 2 °C, 60% relative humidity, 12/12 h light/dark cycle, and provided with food and water ad libitum), and animal health and behavior were daily monitored.

Mice were randomly divided into two groups; namely, lentivirus (lv)-shNC and lv-shSNHG12 groups. The concentration of tumor cells was adjusted to 5 × 107cells/ml, and mice was subcutaneously inoculated with 100 μl cells at the axillary skin of the left forelimb. Tumors were measured every three days, and tumor size was calculated as follows: V=lw2/2. On Day 21, mice were euthanized using an intraperitoneal injection of 150 mg/kg pentobarbital sodium, and tumors were collected. The maximum diameter of tumors recorded in the present study did not exceed 15 mm. A combination of methods was used to ensure the death of the mice, including a firm toe pinch, a lack of visible respiration, a lack of digitally palpable heartbeat or respiration, gray mucous membranes and the loss of corneal reflex. The present study was approved by the Animal Care Board of The First Affiliated Hospital of Nanjing Medical University.

### H&E staining

Tissues were fixed with 4% paraformaldehyde, and subsequently dehydrated, embedded and sliced. Tissue sections were dewaxed with xylene and a gradient concentration of alcohol, and stained with hematoxylin for 3 min. Following washing with H_2_O, tissues were counterstained with ammonia, washed with H_2_O and stained with eosin for 1 min. Following dehydration and blocking, tissue sections were observed under a microscope (Nikon Corporation).

### Immunohistochemical analysis

Tissue sections were fixed in paraffin, deparaffinized and immersed in EDTA buffer. Following blocking with 1% BSA, tissue sections were incubated with primary antibodies against Ki-67 (1:1,000; cat. no. ab92742; Abcam) and SLC7A11 (1:1,000; cat. no. ab307601; Abcam). Subsequently, sections were incubated with secondary antibodies, and immunostaining was performed using streptavidin-peroxidase and diaminobenzidine (Beyotime Institute of Biotechnology), following the manufacturer’s instructions. Tissue sections were analyzed using a microscope (magnification, x400; Nikon Corporation).

### Bioinformatics analysis

The mRNA/lncRNA expression profiles (GSE191209) were downloaded from the publicly available database Binding sites between miR-326 and SNHG12/SLC7A11 were predicted using StarBase (version, 3.0; https://starbase.sysu.edu.cn/). In addition, StarBase 3.0 and GEPIA (http://gepia.cancer-pku.cn/index.html) were used to analyze miR-326/SNHG12/SLC7A11 expression levels in NSCLC cells. M2 macrophage infiltration was analyzed using TIMER (version, 2.0; http://timer.comp-genomics.org/).

All data are presented as the mean ± standard deviation. Data were analyzed using SPSS software (version, 22.0; IBM Corp.). Differences between two groups were analyzed using a Student’s t-test, and differences between multiple groups were analyzed using one-way ANOVA followed by Tukey’s post hoc test. *p* < .05 was considered to indicate a statistically significant difference.

## Results

### NSCLC cell-derived sEVs induce M2 macrophage polarization

**Figure 1.**
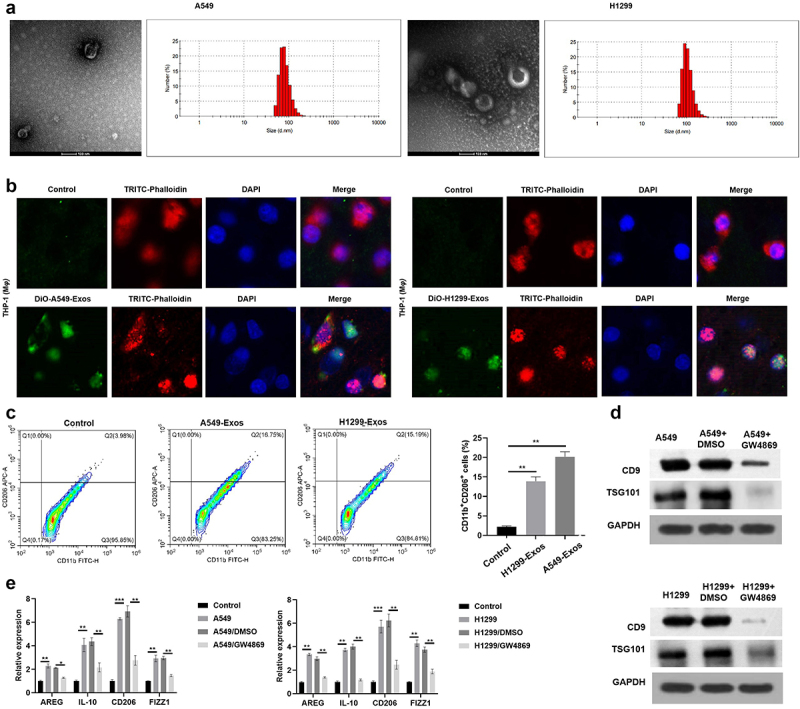
NSCLC cell derived sEVs promotes M2 polarization.

SEVs play essential roles in intercellular communications.^22^ Notably, cancer cell-derived sEVs modify both local and distant microenvironments,^23^ impacting TAM2 polarization. Therefore, the present study aimed to determine the potential association between NSCLC cell-derived sEVs and TAM2 polarization. Results of TEM and NTA analyses demonstrated that the diameter of NSCLC cell-derived sEVs was ~100 nm (Figure 1(a)). Subsequently, THP1 cells were treated with DiO-labeled NSCLC cell-derived sEVs, and the results revealed that DiO intensity was significantly enhanced following treatment with NSCLC cell-derived sEVs, compared with the control group (Figure 1(b)). Moreover, following treatment with NSCLC cell-derived sEVs, the number of CD11b+ CD206+ macrophages was significantly increased (Figure 1(c)). To further verify the role of NSCLC cell-derived sEVs in TAM2 polarization, THP1 cells were treated with the sEV inhibitor, GW4869. As displayed in Figure 1(d), CD9 and TSG101 protein expression levels were markedly decreased following treatment with GW4869. These results suggested that treatment with GW4869 led to the suppression of NSCLC cell-derived sEVs. Moreover, GW4869 treatment inhibited the release of M2 markers, such as AREG, IL-10, CD206 and FIZZ1 (Figure 1(e)). Collectively, results of the present study suggested that NSCLC cell-derived sEVs promoted TAM2 polarization.

(a) NSCLC derived sEVs verified using TEM and NTA assay. (b) The transmission of NSCLC cell derived sEVs. (c) The number of CD11b^+^CD206^+^ macrophages treatment with NSCLC cell-derived sEVs (d) The protein expression of sEVs markers in NSCLC cells after GW4869 treatment. (e) The expression of M2 macrophages markers determined by RT-qPCR. **p < .01, ***p < .001. Control: Cells not treated with sEVs

### TAM2 polarization promotes the proliferation, migration and invasion of NSCLC cells

**Figure 2.**
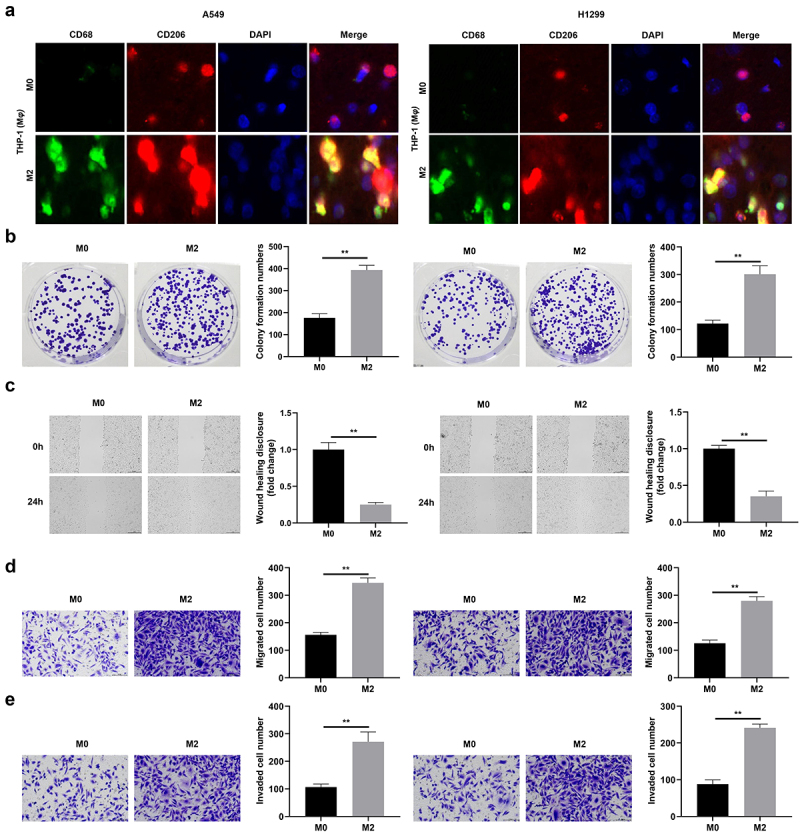
M2 polarization promotes the proliferation, migration, and invasion of NSCLC cells.

M2 macrophage polarization is associated with pro-tumorigenic outcomes.^24^ Thus, the present study aimed to investigate the potential effects of TAM2 on the behavior of NSCLC cells. In the present study, cells were co-cultured with M0 and M2 macrophages, and the results demonstrated that the fluorescence intensity of CD68 and CD206 was markedly increased in the group cultured with M2 macrophages (Figure 2(a)). Moreover, co-culturing with TAM2 significantly increased the colony formation of A549 and H1299 cells (Figure 2(b)), and A549 and H1299 cell migration was markedly increased in the M2 macrophage group (Figure 2(c,d)). Results of the present study also demonstrated that TAM2 polarization significantly increased the invasion of A549 and H1299 cells (Figure 2(e)). Collectively, these results suggested that TAM2 polarization markedly promoted the aggressiveness of NSCLC.

(a) The fluorescence intensity of CD68 and CD206 in the group cultured with M2 macrophages. (b) The cell proliferation of NSCLC cells determined by colony formation assay. (c) The migration of NSCLC cell detected by wound healing assay. (d) The migration of NSCLC cell detected by transwell assay. (e) The invasion of NSCLC cell detected by transwell assay. **p < .01.

### TAM2 polarization suppresses the ferroptosis of NSCLC cells

**Figure 3.**
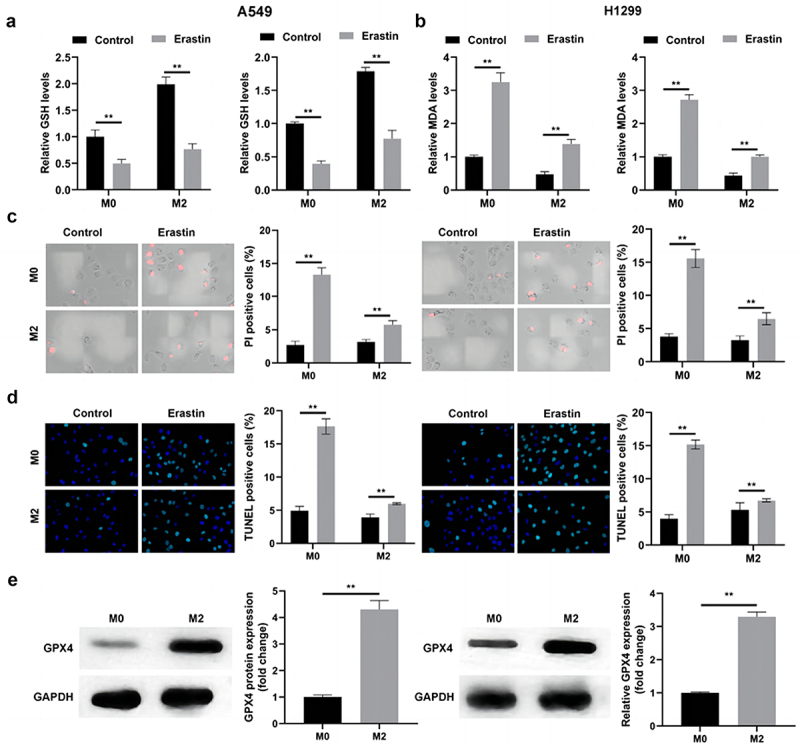
M2 polarization suppresses the ferroptosis of NSCLC cells.

TAM2 polarization suppresses innate immunity and tumoral ferroptosis.^25^ Thus, the potential effects of TAM2 polarization on Erastin-induced ferroptosis were determined in the present study. As displayed in Figure 3(a,b), Erastin markedly increased the release of MDA and reduced the release of GSH. However, these results were reversed following co-culturing with TAM2. Results of the present study also revealed that the Erastin-induced increase in the number of PI-positive cells was significantly decreased in the M2 macrophage group (Figure 3(c)). Notably, these results were consistent with those obtained using the TUNEL assay. As displayed in Figure 3(d), co-culturing with TAM2 markedly decreased the number of TUNEL-positive cells, and increased GPX4 protein expression levels (Figure 3(e)). As GPX4 is a ferroptosis suppressor, these results suggested that TAM2 polarization may promote the survival of NSCLC cells.

(a) The release level of GSH in NSCLC cells with or without Erastin. (b) The release level of MDA in NSCLC cells with or without Erastin. (c) Cell death in NSCLC cells with or without Erastin detected by PI staining. (d) Cell death in NSCLC cells with or without Erastin detected by TUNEL assay. (e) GPX4 protein expression determined by western blot. **p < .01. Control: Cells not treated with Erastin.

### SNHG12 promotes TAM2 polarization

**Figure 4.**
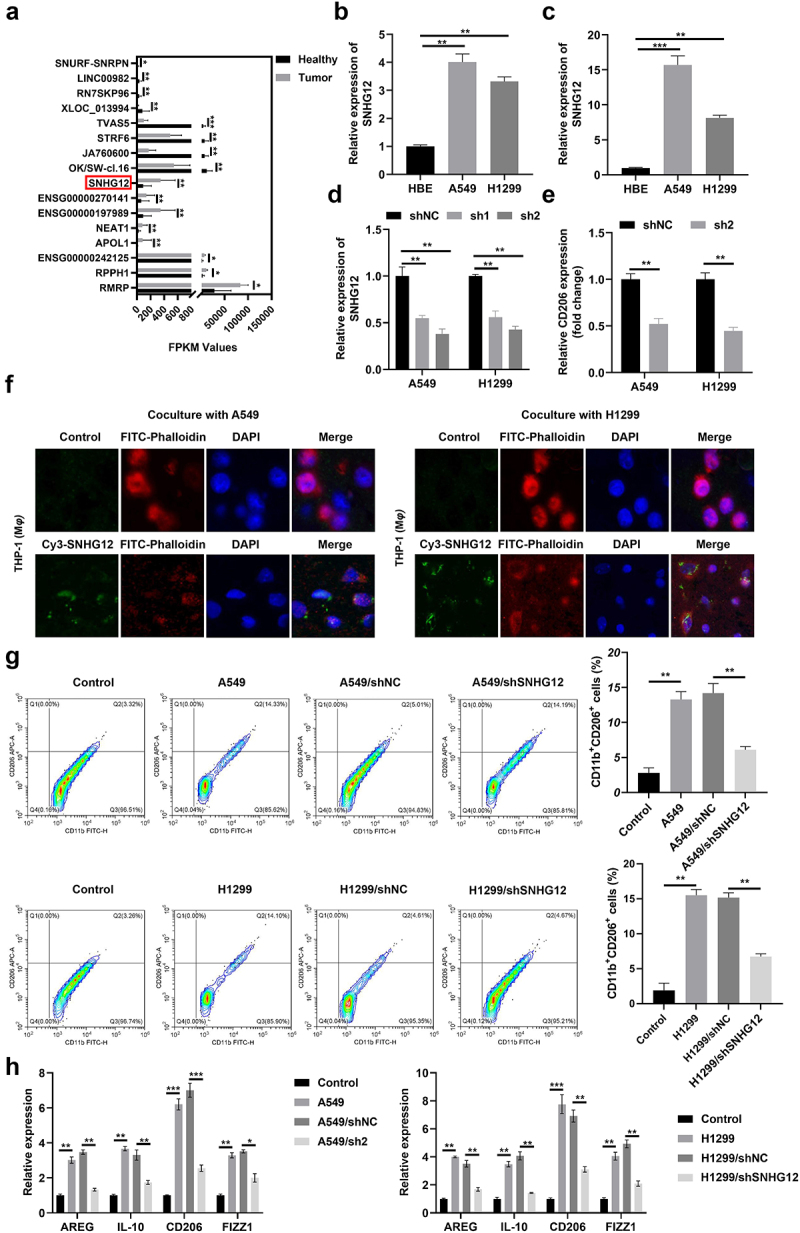
SNHG12 promotes the M2 polarization.

SEVsLncRNAs contain high levels of sEVs and the abnormal expression of lncRNAs is closely associated with tumorigenesis. Thus, the present study aimed to examine differentially expressed lncRNAs derived from plasma sEVs obtained from patients with NSCLC. Differentially expressed lncRNAs are displayed in Figure 4(a). Results of the present study revealed that the levels of SHNG12, SNHG3, APOL1 and NEAT1 were markedly increased in NSCLC cells and tumor cell-derived sEVs (Figure 4(b,c); Figure S1). Moreover, SNHG12 expression levels differed between tumor cells and sEVs (Figure 4(b,c)). To further verify the potential role of SNHG12 in NSCLC, the aforementioned lncRNAs were examined in NSCLC cells following transfection with shRNA. Results of the present study revealed that SNHG12 expression was markedly decreased in NSCLC cells (Figure 4(d)). In addition, CD206 expression was markedly decreased following transfection with shSHNG12, and shNEAT1 knockdown markedly decreased the expression of CD206 (Figure 4(e) and Figure S2). Thus, SHNG12 was selected for use in subsequent experiments. To verify the potential role of SHNG12 in TAM2 polarization, THP1 cells were co-cultured with A549 and H1299 cells transfected with Cy3-SNHG12. Results of the fluorescence analysis revealed the presence of Cy3-SNHG12 in the co-culture group (Figure 4(f)), indicative of the successful transfection with SNHG12 in THP1 cells. Moreover, SNHG12 knockdown significantly reduced the number of CD11b^+^ CD206^+^ macrophages (Figure 4(g)), and reduced the release of AREG, IL-10, CD206 and FIZZ1 (Figure 4(h)). Collectively, these findings suggested that NSCLC cell-derived SNHG12 may promote TAM2 polarization.

(a) The abnormal expressed lncRNAs in the plasma from tumor tissues of NSCLC patients. (b) SNHG12 expression in NSCLC cells determined by RT-qPCR. (c) The expression of SNHG12 in NSCLC cell derived sEVs. (d) Transfection efficiency of shSNHG12 determined by RT-qPCR. (e) The mRNA expression of CD206 detected by RT-qPCR. (f) The transmission of NSCLC cell derived sEVs determined by immunofluorescence. (g) The number of CD11b+CD206+ macrophages after sEVs with lower SNHG12 treatment determined by flow cytometry. (h) The release of M2 macrophages markers determined by RT-qPCR. sh1 and sh2 are short hairpin RNA. **p < .01, ***p < .001. Control: Cells not treated with sEVs.

### SNHG12 knockdown suppresses NSCLC cell proliferation, migration and invasion, and promotes ferroptosis

**Figure 5.**
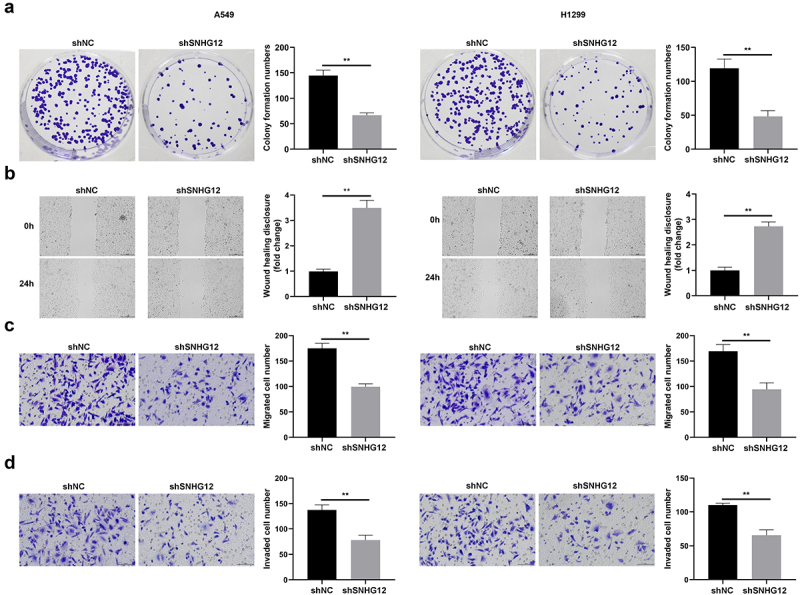
SNHG12 knockdown inhibits the proliferation, migration and invasion of NSCLC cells.

In the present study, the potential effects of SNHG12 knockdown on the cellular functions of NSCLC cells were investigated. As displayed in Figure 5(a), colony formation of A549 and H1299 cells was markedly suppressed following SNHG12 knockdown. Moreover, SNHG12 knockdown significantly suppressed the migration and invasion of A549 and H1299 cells (Figure 5(b-d)).

(a) The cell proliferation of NSCLC cells determined by colony formation assay. (b) The migration of NSCLC cell detected by wound healing assay. (c) The migration of NSCLC cell detected by transwell assay. (d) The invasion of NSCLC cell detected by transwell assay. **p < .01.

**Figure 6.**
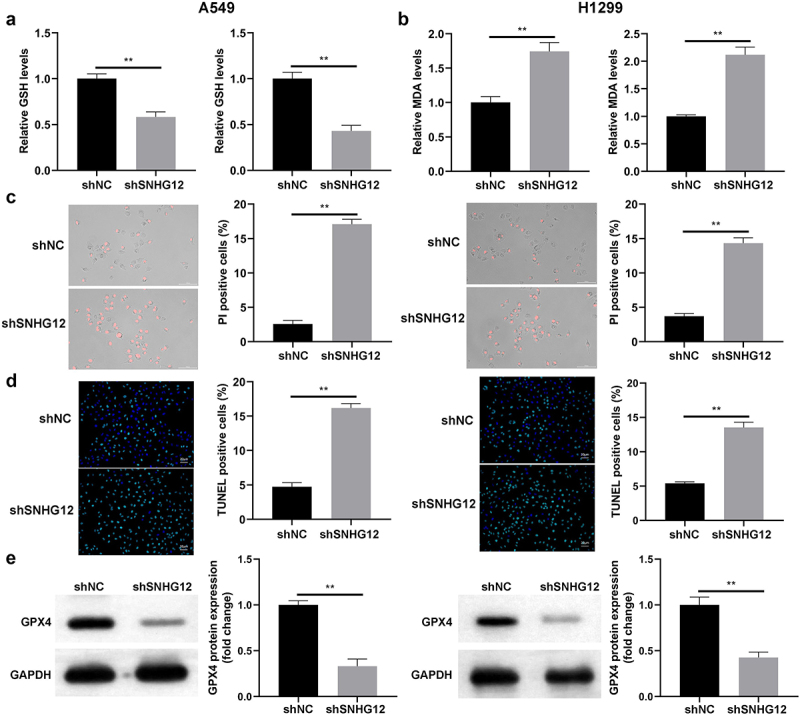
SNHG12 knockdown promotes the ferroptosis of NSCLC cells.

Results of the present study also revealed that SNHG12 knockdown significantly increased the release of MDA and decreased the release of GSH (Figure 6(a,b)). SNHG12 knockdown markedly increased the number of PI- and TUNEL-positive cells (Figure 6(c,d)), and decreased GPX4 protein expression (Figure 6(e)). Collectively, these findings suggested that SNHG12 knockdown may suppress the malignant behavior of NSCLC cells.

(a) The release level of MDA in SNHG12 knockdown NSCLC cell and normal NSCLC cell. (b) The release level of GSH in SNHG12 knockdown NSCLC cell and normal NSCLC cell. (c) Cell death of SNHG12 knockdown NSCLC cell and normal NSCLC detected by PI staining. (d) Cell death of SNHG12 knockdown NSCLC cell and normal NSCLC detected by TUNEL assay. (e) GPX4 protein expression of SNHG12 knockdown NSCLC cell and normal NSCLC determined by western blot. **p < .01.

### SNHG12 regulates SLC7A11 via sponging miR-326

LncRNA-mediated upregulation of the ferroptosis suppressor promotes metastasis and evasion in lung cancer. Thus, the expression of well-established ferroptosis suppressors were investigated in the present study using GEPIA. The results demonstrated that CISD1, EMC2, FANCD2, HSPA5, GPX4, HSPB1, SLC5A1 and SLC7A11 were overexpressed in LAUD and LUSC (Figure S3). In addition, results of the present study indicated that FANCD2, GPX4, HSPA5, SLC5A1 and SLC7A11 mRNA expression levels were markedly increased in NSCLC cells, suggesting that the xCT system may play a role in the development of NSCLC (Figure S4a). The activation of xCT contributes to ferroptosis resistance in NSCLC cells and suppresses immune activation. To verify the downstream target of SNHG12, cells were transfected with shSNHG12. Results of the present study demonstrated that SNHG12 knockdown significantly decreased the mRNA expression of GPX4 and SLC7A11 (Figure S4b). Moreover, CD206 mRNA expression levels were markedly decreased following transfection with shSLC7A11; however, CD206 expression was not altered following transfection with shGPX4 (Figure S5a). In addition, results of the present study revealed that SLC7A11 expression was positively associated with the activation of TAM2 and its infiltration (Figure S5b).

**Figure 7.**
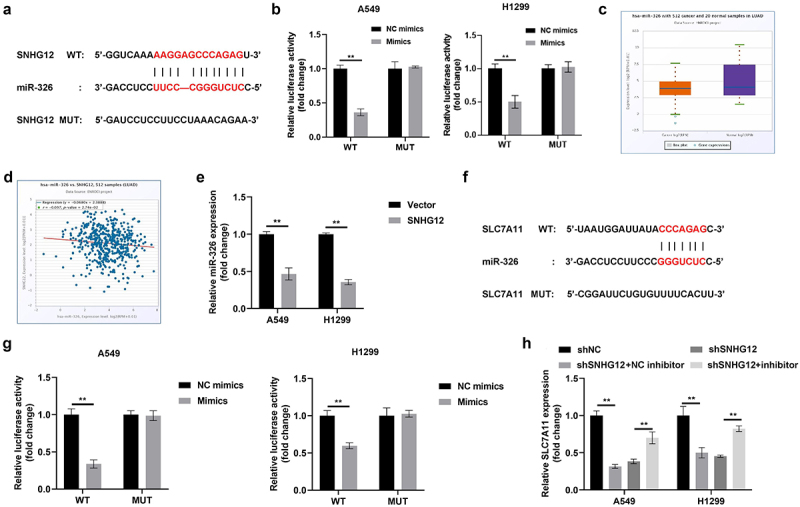
SNHG12 regulates miR-326/SLC7A11 axis.

LncRNAs regulate cell function via ceRNA mechanisms, impacting the expression of specific genes through targeting miRNAs. Results of a previous study revealed that miR-326 is involved in the regulation of NSCLC and is regulated by SNHG12. Thus, the potential binding sites between SNHG12 and miR-326 were predicted using StarBase 3.0 (Figure 7(a)). Results of the present study revealed that luciferase activity was markedly decreased in A549 and H1299 cells transfected with SNHG12 WT and miR-326 mimics (Figure 7(b)). Moreover, miR-326 expression levels were significantly decreased in patients with LAUD (Figure 7(c)), and these levels were negatively associated with miR-326 (Figure 7(d)). Notably, miR-326 expression was markedly decreased following transfection with SNHG12 (Figure 7(e)). As displayed in Figure 7(f), miR-326 exhibited binding sites with SLC7A11, and these binding sites were verified using a dual-luciferase reporter assay (Figure 7(g)). In addition, results of the present study revealed that SLC7A11 mRNA expression levels were significantly increased following transfection with SNHG12, and these effects were reversed following transfection with the miR-326 mimic (Figure 7(h)). Collectively, these findings suggested that SNHG12 increased SLC7A11 expression via sponging miR-326.

(a) The potential binding sites between SNHG12 and miR-326 predicted by Starbase3.0. (b) The binding sites of SNHG12 and miR-326 verified by luciferase assays. (c) SNHG12 expression in NSCLC patients analyzed by Starbase3.0. (d) The correlation between SNHG12 and miR-326 expression analyzed by Starbase3.0. (e) miR-326 expression detected by RT-qPCR. (f) The potential binding sites between miR-326 and SLC7A11 predicted by Starbase3.0. (g) The binding sites of miR-326 and SLC7A11 verified by luciferase assay. (h) SLC7A11 mRNA expression determined by RT-qPCR. **p < .01.

### NSCLC cells adapt to TAM2 polarization in the tumor microenvironment via SNHG12-mediated SLC7A11

**Figure 8.**
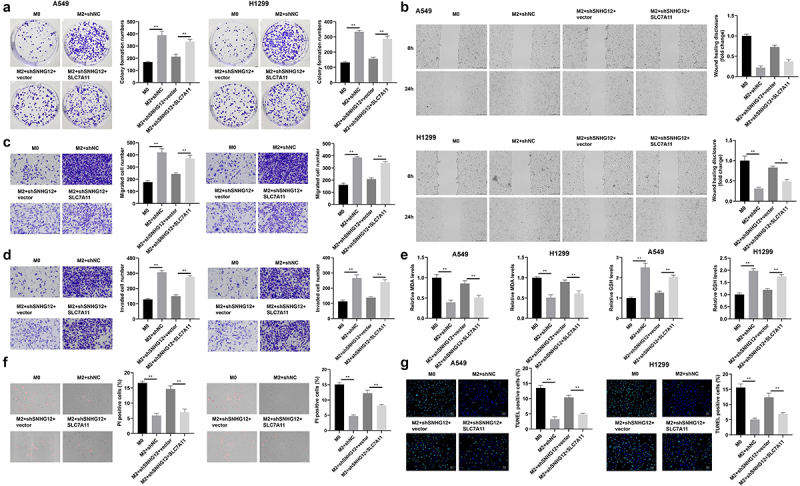
NSCLC cells adapt to TAM2 polarization in the tumor microenvironment via SNHG12-mediated SLC7A11 pathway activation.

**Figure 9.**
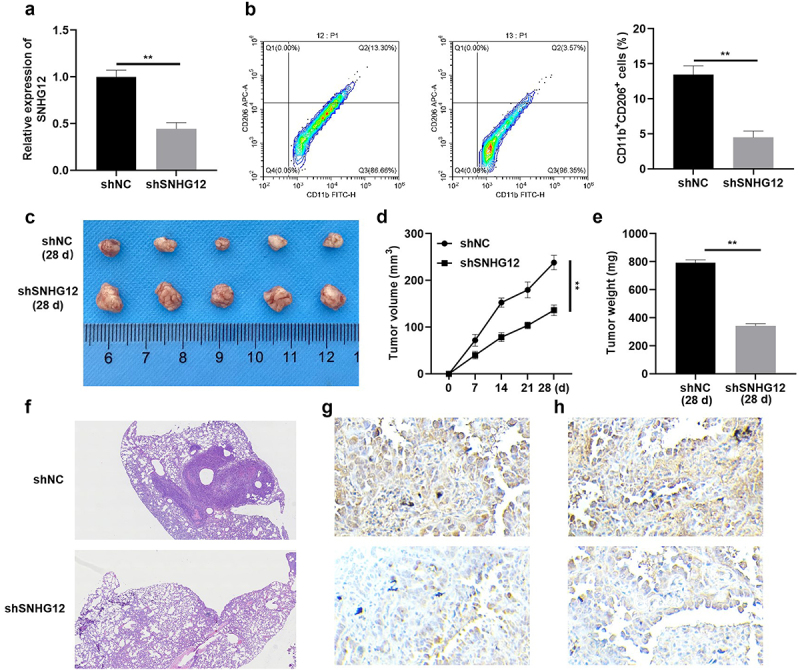
SNHG12 knockdown suppresses tumor growth and metastasis of NSCLC.

In the present study, TAM2 filtration was analyzed using TIMER (Figure 8(a)), and rescue assays were performed to further verify the role of SLC7A11 in NSCLC. Results of the present study revealed that the number of NSCLC colonies was significantly increased in the M2 macrophage group; however, this was markedly decreased following SNHG12 knockdown, and alleviated following SLC7A11 expression (Figure 9(b)). Moreover, SLC7A11 overexpression promoted the migration (Figure 8(c)) and invasion (Figure 8(d)) of A549 and H1299 cells. Results of the present study also revealed that SLC7A11 significantly reduced the release of MDA and increased the release of GSH (Figure 8(e)). Compared with the shSNHG12 group, SLC7A11 overexpression markedly decreased the number of PI- (Figure 8(f)) and TUNEL-positive cells (Figure 8(g)). Collectively, these findings suggested that SNHG12-mediated SLC7A11 upregulation promoted the adaptation of NSCLC cells to TAM2 polarization in the tumor microenvironment.

(a) The correlation between TAM2 filtration analyzed by TIMER. (b) The cell proliferation of NSCLC cells determined by colony formation assay. (c) The migration of NSCLC cell detected by wound healing assay. (d) The invasion of NSCLC cell detected by transwell assay. (e) The release level of MDA and GSH. (f) Cell death detected by PI staining. (g) Cell death detected by TUNEL assay. **p < .01.

(a) The transfection efficiency of shSNHG12 determined by RT-qPCR. (b) The number of CD11b^+^CD206^+^ macrophages determined by flow cytometry. (c) The tumor size, (d) volume and (e) weight. (f) Histological analysis of lung metastasis detected by HE staining. The expression of Ki-67 (g) and SCL7A11 (h) determined by immunohistochemistry. **p < .01.

### SNHG12 suppresses tumor growth and metastasis in vivo

To further verify the role of SNHG12 in NSCLC, A549 cells transfected with lv-shSNHG12 or lv-shNC were injected into mice. Results of the present study demonstrated that SNHG12 knockdown significantly decreased tumor size (Figure 9(a)), volume (Figure 9(b)) and weight (Figure 9(c)). Moreover, SNHG12 expression was significantly reduced following transfection with shSNHG12, indicative of successful transfection in A549 and H1299 cells (Figure 9(d)). Results of the present study revealed that SNHG12 knockdown reduced the number of CD11b^+^ CD206^+^ macrophages (Figure 9(e)), and significantly reduced the number of nodules (Figure 9(f)). Moreover, SNHG12 knockdown reduced Ki-67 and SLC7A11 expression levels (Figure 9(g,h)). Collectively, these findings suggested that SNHG12 knockdown may suppress the aggressiveness of NSCLC *in vivo*.

### SNHG12 exhibits potential as a biomarker for NSCLC

**Figure 10.**
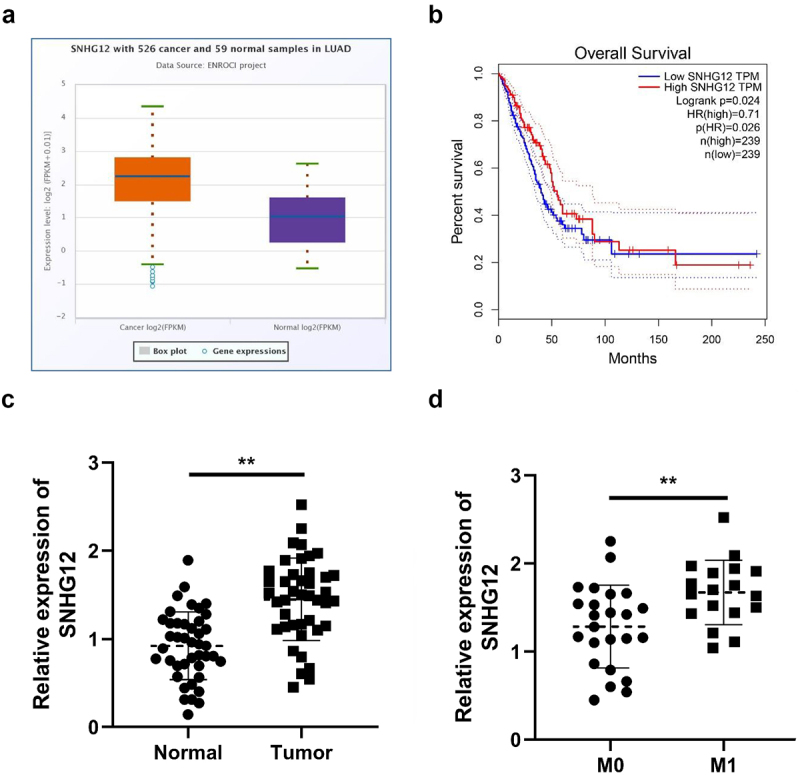
SNHG12 can be a potential biomarker for NSCLC.

To further verify the role of SNHG in NSCLC, SNHG12 expression levels were investigated in patients with NSCLC. Results of the present study revealed that SNHG12 was overexpressed in patients with LUAD (Figure 10(a)). Notably, low SHNG12 expression levels were associated with favorable long-term survival rates in patients with LUAD (Figure 10(b)), and SNHG12 expression was significantly increased in patients with NSCLC (Figure 10(c)). Results of the present study also indicated that SNHG12 expression was significantly increased in patients with NSCLC and lymph node metastasis (Figure 10(d)).

(a) SNHG12 expression analyzed by Starbase3.0. (b) The correlation between SNHG12 expression and the survival rates of LUAD patients analyzed by GEPIA. (c) SNHG12 expression determined by RT-qPCR. (d) SNHG12 expression determined by RT-qPCR. **p < .01.

## Discussion

Results of the present study revealed that SNHG12 may function as an oncogene in NSCLC. NSCLC-cell derived SNHG12 promoted TAM2 polarization in the tumor microenvironment, which induced the proliferation, migration and invasion of NSCLC cells. Moreover, SNHG12-mediated TAM2 polarization suppressed the ferroptosis of NSCLC cells, and SNHG12 sponged miR-326 to upregulate SLC7A11. Results of the present study also demonstrated that SLC7A11 promoted the adaptation of NSCLC cells to TAM2 polarization in the tumor microenvironment. Therefore, the SNHG12/miR-326/SLC7A11 axis may exhibit a potential as a novel target for the treatment of NSCLC.

Tumor-secreted sEVs are critical mediators of intercellular communication between tumor cells and stromal cells in local and distant microenvironments.^26^ SEVs orchestrate multiple systemic pathophysiological processes to induce pre-metastatic niche formation and subsequent metastasis.^27^ Nucleic acids, including miRNA, lncRNA and circRNA, are rich within sEVs.^28^ sEVsResults of the present study revealed that NSCLC cell-derived lncRNA, SNHG12, promoted M2 macrophage polarization. Notably, transformation into TAMs reshapes the tumor microenvironment and pre-metastasis niches.^29^ Following co-culturing with M2 macrophages and NSCLC cells, NSCLC cell proliferation, migration and invasion were increased.

LncRNAs play a crucial role in tumorigenesis, functioning as oncogenes or anti-tumor genes in various tumors, including lung cancer.^7–12^ Results of a previous study revealed that lncRNA LINC00115 enhanced the proliferation, migration and invasion of lung cancer cells.^30^ However, lncRNA BRCAT54 promoted apoptosis and inhibited the tumorigenesis of NSCLC.^31^ In addition, lncRNA SNHG12 acts as an oncogene in renal cell carcinoma, colon cancer and NSCLC;^12,32,33^ however, the specific role of SNHG12 in esophageal squamous cell carcinoma (ESCC) remains to be fully elucidated. Results of a previous study revealed that low SNHG12 expression levels in patients with ESCC are associated with poor clinical outcomes.^34^ In CD133-ESCC cells, SNHG12 overexpression promoted the proliferation and stemness of ESCC cells.^35^ Collectively, these results indicated that SNHG12 may act as an oncogene or tumor suppressor in ESCC, and the role of SNHG12 may vary depending on cell subtypes and underlying molecular mechanisms. Thus, further investigations are required to determine the specific role of SNHG12 in NSCLC. Results of the present study revealed that high SNHG12 expression levels are associated with TAM2 infiltration, lymph node metastasis and poor long-term survival rates in patients with NSCLC. These findings suggested that SNHG12 may function as an oncogene in NSCLC, which is consistent with the results obtained by Wang *et al*.^12^ Results of previous studies demonstrated that SNHG12 participates in the tumorigenesis of NSCLC via the regulation of tumor cell function and reshaping of the tumor microenvironment.^11,12^ In the present study, NSCLC cell-derived SNHG12 promoted TAM2 polarization, leading to increased NSCLC cell proliferation, migration and invasion, and suppression of ferroptosis. Therefore, SNHG12 may play a role in modifying the tumor microenvironment, leading to the increased growth of tumor cells and metastasis. Notably, SNHG12 knockdown suppressed NSCLC tumor growth and metastasis both *in vivo* and *in vitro*, highlighting the potential of SNHG12 as a therapeutic target in NSCLC.

Results of previous studies revealed that lncRNAs may regulate cellular functions via regulation of the miRNA/mRNA axis.^8–10^ For example, cancer-associated fibroblast-derived OIP5-AS1 promoted immune invasion in NSCLC via regulation of the miR-142-5p/PD-L1 axis.^36^ In addition, lncRNA HOST2 enhanced the chemoresistance of NSCLC cells via binding to the miR-621/SYF2 axis.^37^ Collectively, these findings suggested that lncRNAs may sponge miRNAs to upregulate the expression of oncogenes, functioning as ceRNA. Results of the present study demonstrated that SNHG12 sponged miR-326 to induce the upregulation of SLC7A11. Notably, miR-326 acts as a tumor suppressor in lung cancer.^38^ Results of our previous study revealed that miR-326 suppresses the proliferation and migration of lung cancer cells, and promotes cell cycle arrest and apoptosis.^39,40^ These results were consistent with those obtained by Zhang *et al*.^38^ In addition, results of a previous study revealed that miR-326 suppressed tumor cell stemness and M2 macrophage polarization, and alleviated chemoresistance in NSCLC.^41–43^ Results of the present study revealed that SNHG12 negatively regulated miR-326 expression, highlighting that the sponging of miR-326 by SNHG12 may inhibit the anti-tumor activity of miR-326.

SLC7A11 plays a role in importing cystine for glutathione biosynthesis and antioxidant defense.^44^ Notably, SLC7A11 acts as an oncogene in numerous cancers via antioxidant activity, suppression of excessive lipid peroxidation^45^ and regulation of metabolic reprogramming in the tumor microenvironment. SLC7A11 promotes the synthesis of cysteine from extracellular cystine, which is primarily required for tumor cells.^46^ Moreover, SLC7A11 participates in regulating glucose metabolism and the TCA cycle, supporting cancer cell survival and distant metastasis.^47,48^ Results of the present study revealed that SLC7A11 overexpression promoted the release of GSH and suppressed oxidative stress. Moreover, SNHG12-mediated upregulation of SLC7A11 suppressed ferroptosis and promoted the adaptation of NSCLC cells to TAM2 polarization in the tumor microenvironment.

Results of a previous study demonstrated that the ferroptosis of tumor cells promotes TAM2 polarization for development of the tumor-killing M1 macrophages.^49^ Notably, genes associated with the ferroptosis pathway are involved in cell reprogramming; for example, SCL7A11 and GPX4 play a key role in lipid peroxidation,^50^ ACSL4 plays a role in fatty acid metabolism^51^ and TFRC, FTH1 and FTL1 play roles in iron homeostasis.^52,53^ However, the abnormal expression of these genes reshapes the tumor microenvironment, leading to reduced innate immunity, and increased tumor immune infiltration and resistance to immunotherapy.^54,55^ Results of the present study revealed that SLC7A11 suppressed the ferroptosis of NSCLC cells, and promoted the adaptation of NSCLC cells to TAM2 polarization in the tumor microenvironment. Moreover, results of the present study revealed that SLC7A11 expression was positively associated with TAM2 infiltration, and e SNHG12 mediated TAM2 polarization and the aggressiveness of NSCLC via SLC7A11 signaling.

In conclusion, results of the present study indicated that SNHG12 may function as an onco-lncRNA in NSCLC. In addition, SNHG12-mediated upregulation of SCL7A11 suppressed tumor cell ferroptosis, and promoted the TAM2 polarization, proliferation, migration and invasion of NSCLC cells via sponging miR-326. Thus, the SNHG12/miR-326/SCL7A11 axis may exhibit potential as a novel target for the treatment of NSCLC.

## Supplementary Material

Supplemental Material

Supplemental Material

Supplemental Material

Supplemental Material

Supplemental Material

## Data Availability

The data that support the findings of this study are available from the corresponding author upon reasonable request.
