# Supplementary figures and images for: Lung cancer cell derived sEVs enhance the metastasis of non-small cell lung cancer via SNHG12/miR-326/SLC7A11 axis
Source: Cancer Biol Ther. 2025 May 26;26(1):2510041. doi: 10.1080/15384047.2025.2510041 (PMC12118444; doi:10.1080/15384047.2025.2510041)

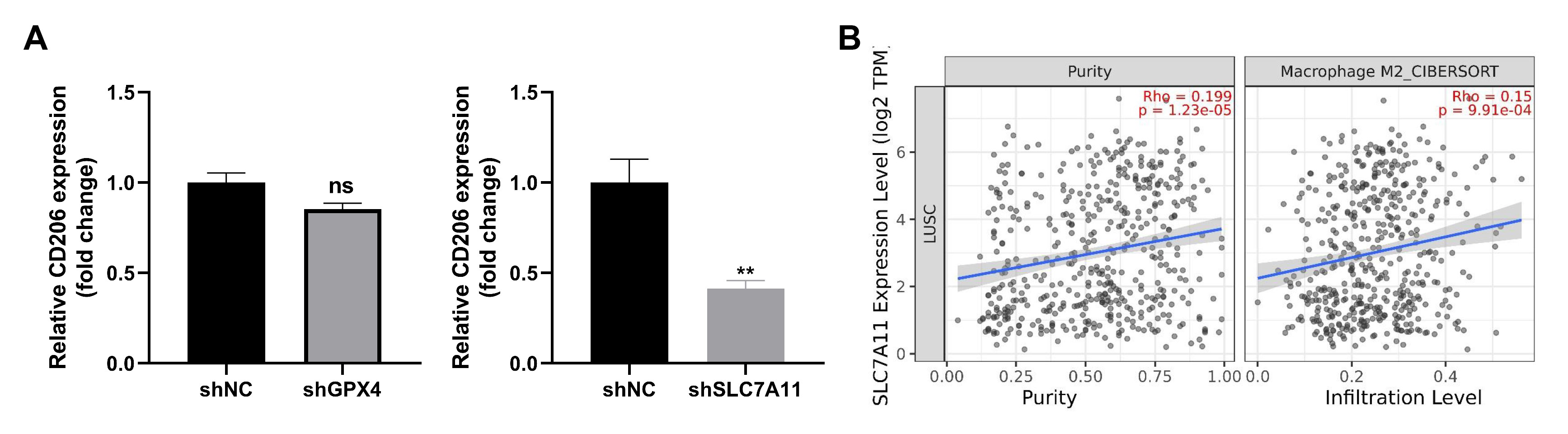

Supplement: Supplemental Material [file KCBT_A_2510041_SM4698.tiff]

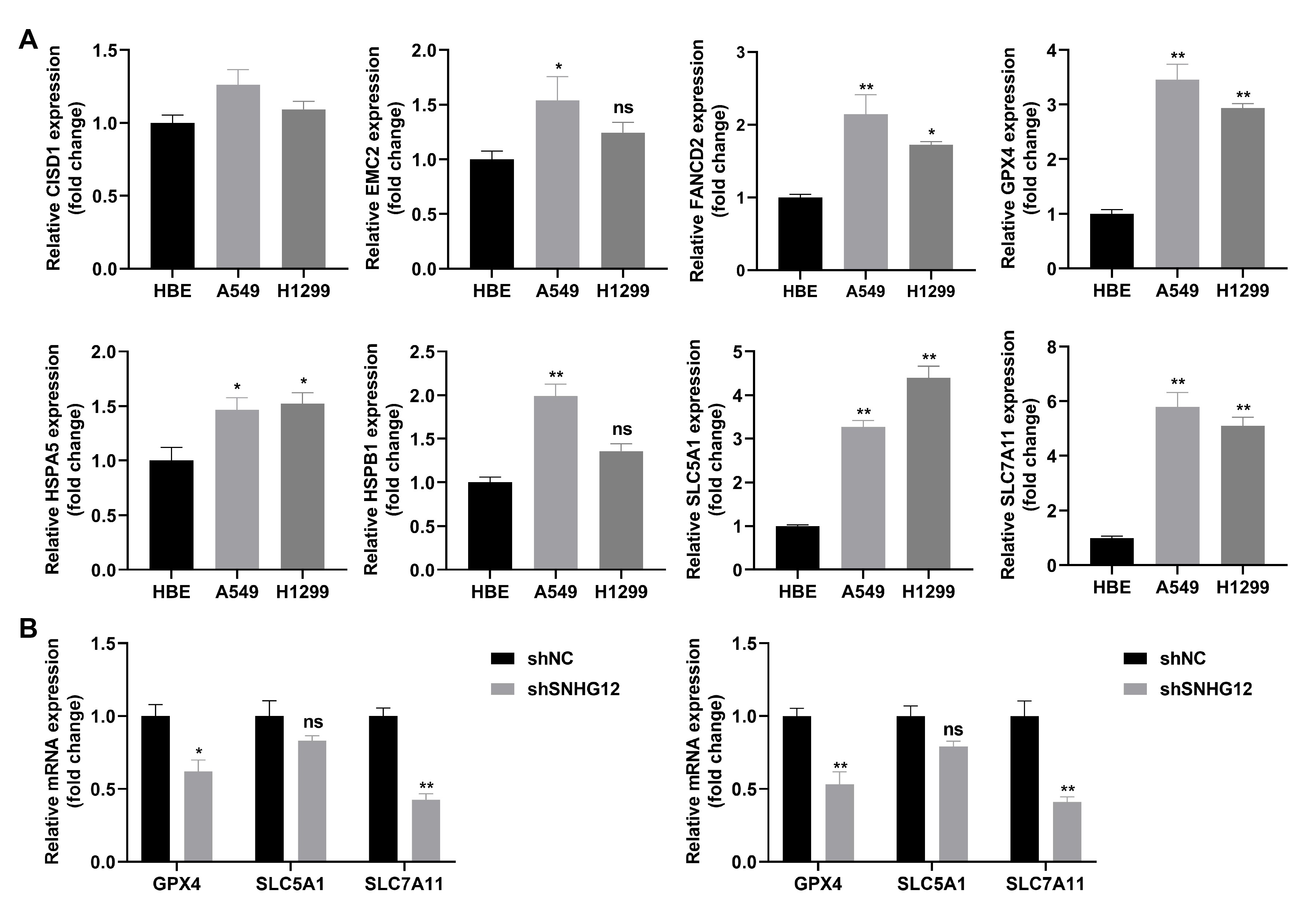

Supplement: Supplemental Material [file KCBT_A_2510041_SM4690.tiff]

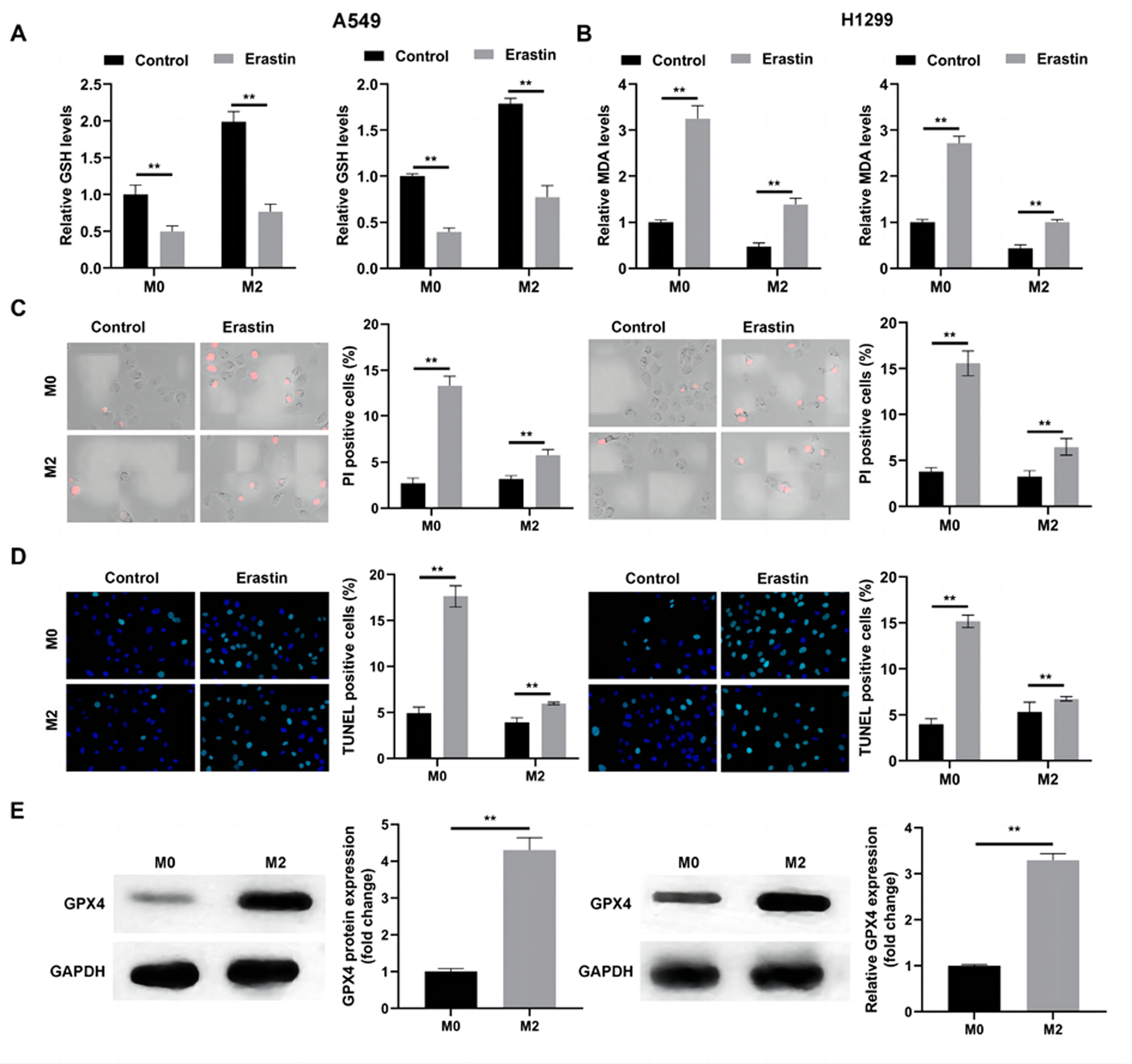

Supplement: Supplemental Material [file KCBT_A_2510041_SM4673.tiff]

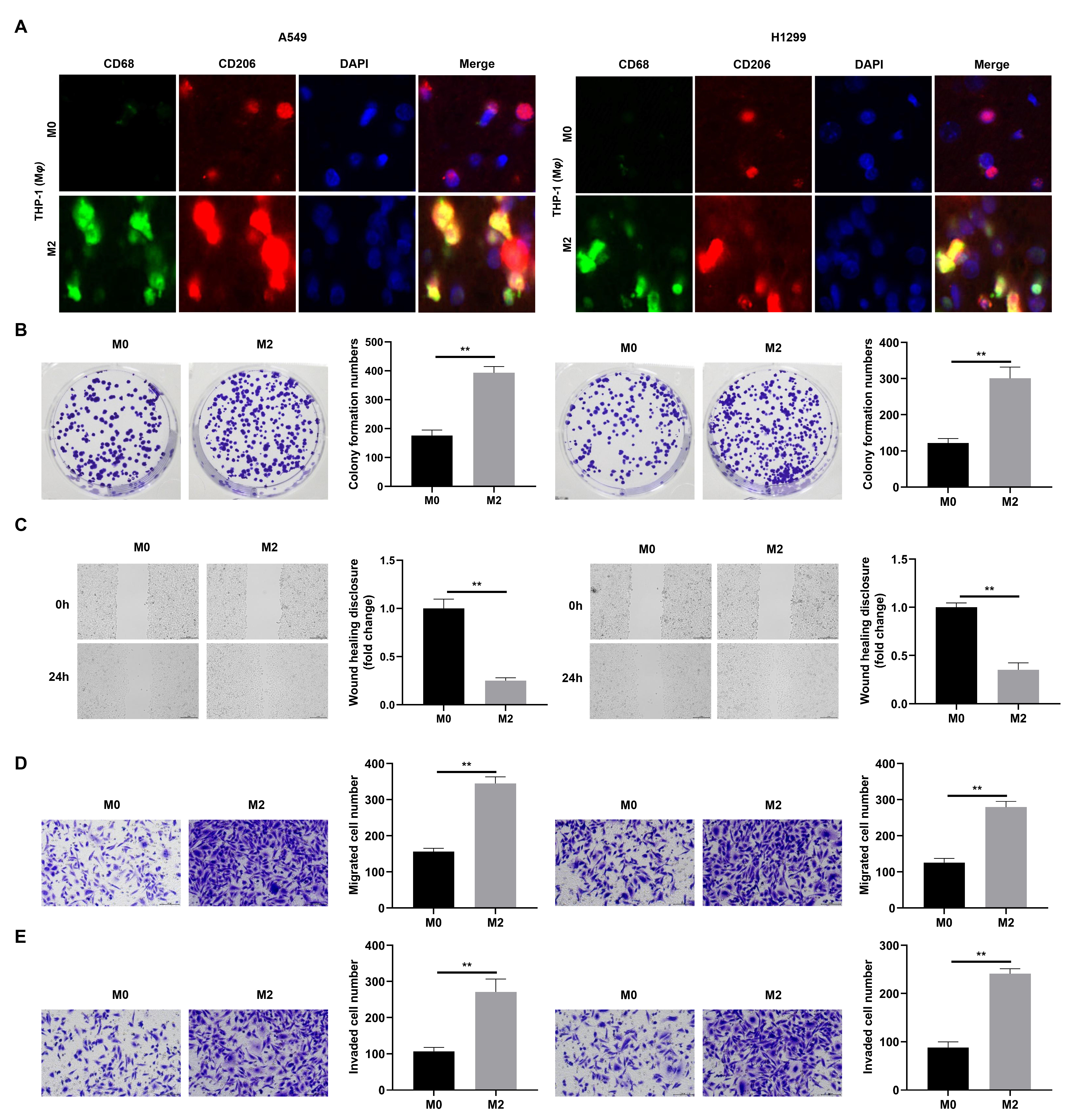

Supplement: Supplemental Material [file KCBT_A_2510041_SM4592.tiff]

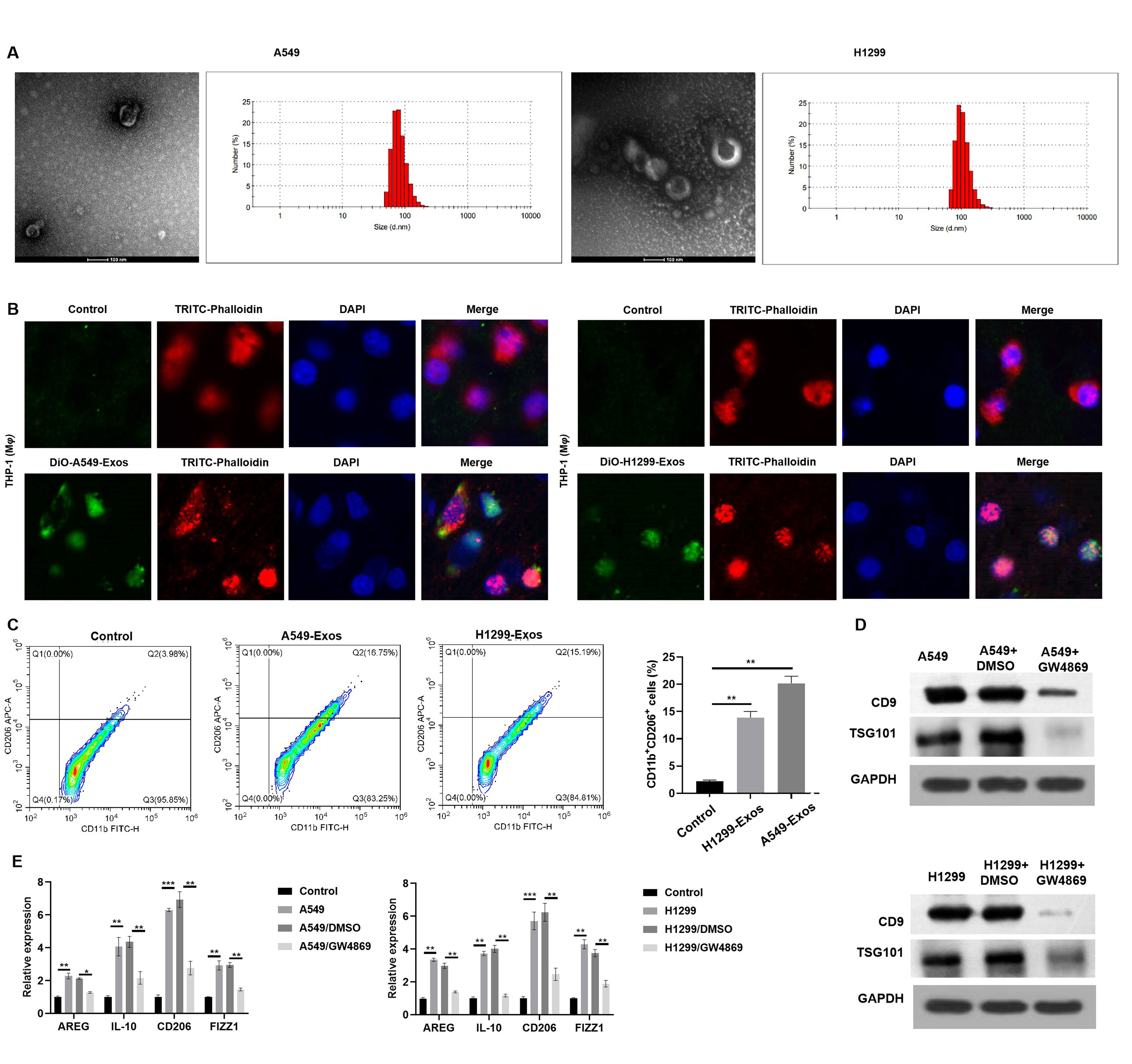

Supplement: Supplemental Material [file KCBT_A_2510041_SM4493.tiff]
